# Combining Multi-Source Remotely Sensed Data and a Process-Based Model for Forest Aboveground Biomass Updating

**DOI:** 10.3390/s17092062

**Published:** 2017-09-08

**Authors:** Xiaoman Lu, Guang Zheng, Colton Miller, Ernesto Alvarado

**Affiliations:** 1 Jiangsu Center for Collaborative Innovation in Geographical Information Resource Development and Application, Nanjing 210023, China; luxmnju@163.com; 2 Jiangsu Provincial Key Laboratory of Geographic Information Science and Technology, International Institute for Earth System Science, Nanjing University, Nanjing 210023, China; 3 School of Environmental and Forest Sciences, University of Washington, Seattle, WA 98195, USA; cwm4@uw.edu (C.M.); alvarado@uw.edu (E.A.)

**Keywords:** process-based model, NPP, AGB, BEPS, ALS

## Abstract

Monitoring and understanding the spatio-temporal variations of forest aboveground biomass (AGB) is a key basis to quantitatively assess the carbon sequestration capacity of a forest ecosystem. To map and update forest AGB in the Greater Khingan Mountains (GKM) of China, this work proposes a physical-based approach. Based on the baseline forest AGB from Landsat Enhanced Thematic Mapper Plus (ETM+) images in 2008, we dynamically updated the annual forest AGB from 2009 to 2012 by adding the annual AGB increment (ABI) obtained from the simulated daily and annual net primary productivity (NPP) using the Boreal Ecosystem Productivity Simulator (BEPS) model. The 2012 result was validated by both field- and aerial laser scanning (ALS)-based AGBs. The predicted forest AGB for 2012 estimated from the process-based model can explain 31% (*n* = 35, *p* < 0.05, RMSE = 2.20 kg/m^2^) and 85% (*n* = 100, *p* < 0.01, RMSE = 1.71 kg/m^2^) of variation in field- and ALS-based forest AGBs, respectively. However, due to the saturation of optical remote sensing-based spectral signals and contribution of understory vegetation, the BEPS-based AGB tended to underestimate/overestimate the AGB for dense/sparse forests. Generally, our results showed that the remotely sensed forest AGB estimates could serve as the initial carbon pool to parameterize the process-based model for NPP simulation, and the combination of the baseline forest AGB and BEPS model could effectively update the spatiotemporal distribution of forest AGB.

## 1. Introduction

The forest ecosystem plays a key role in carbon cycling, gas and matter exchange processes between the biosphere and atmosphere, and accounts for 45–60% carbon stock of a terrestrial ecosystem [1,2,3]. As the accumulated organic matter of a living standing tree or forest stand during a certain period [4], forest AGB works as a pivotal parameter to help quantitatively assess the carbon sequestration capacity of a forest ecosystem [5]. Thus, it is necessary to dynamically monitor the spatiotemporal distribution of the forest AGB and its annual increment [6,7] for studying the forest ecosystem carbon cycle and understanding impacts of global climate changes.

So far, three kinds of methods have been proposed to estimate the forest AGB, including national forest inventory (NFI)-based methods, remotely sensed data-based methods and process model-based methods.

Field-based methods can observe accurate parameters such as tree height and diameter at breast height (DBH), which relate closely with AGB estimation. However, these methods mostly focus on some of the representative wood—rather than all forest biomass—and are always labor intensive and time consuming. Although the remote sensing-based methods have advantages of large-coverage and short revisitation period, they cannot observe tree height and DHB directly. In most cases, the optical remotely sensed-based methods retrieve LAI and coverage degree based on canopy reflectance from sensors, then estimate the AGB. However, given that the canopy reflectance is influenced by both overstory and background vegetation, the remotely sensed-based AGB is always overestimated or underestimated for sparse or dense forests, respectively. The ALS-based methods can retrieve tree height information, but have difficulty in obtaining DHB.

Combining field data and optical remotely sensed data for mapping forest AGB is a more ideal way at the landscape level [5,8,9,10,11,12,13,14]. For example, Zheng et al. [4] obtained the spatial distribution map of forest AGB in Wisconsin (USA) using Landsat 7 ETM+ data combined with NFI data. The biomass for boreal forest was estimated by Muukkonen et al. [14] using ASTER satellite data combined with standwise NFI data. Nevertheless, restricted by the characteristics of optical imaging, only two dimensional (2-D) structural information of land surface objects can be captured by satellite sensors. This shortcoming could not be overcome until the emergence of light detection and ranging (lidar) by the active scanning system such as ALS.

The lidar-based point cloud data generated by ALS have shown great potential to effectively improve the accuracy of retrieving biophysical parameters of forest canopies from three dimensional (3-D) perspective [15,16,17,18,19,20,21]. For instance, Siberian timber volume was estimated by combining Moderate Resolution Imaging Spectroradiometer (MODIS) and ICESat/GLAS data [15]. Hauglin et al. [16] developed an approach to estimate the single tree branch biomass of Norway spruce using ALS data. Although the lidar-based method does have relatively high precision when applied to calculate the forest AGB, it is not the most ideal method to be used at national or regional scales because of the expensive cost of ALS data acquisition and the unavailable space-borne lidar systems since the ICESat mission (2003–2009) termination [21]. Therefore, in consideration of the pros and cons of ALS data, they were only used as validation data in this study.

In the past few decades, physical process-based ecological models [22,23,24], such as the BEPS model [24], have been used to simulate key physiological processes including both carbon and water cycles at national or regional scales. Based on the interaction mechanisms of the electromagnetic waves and vegetation, the BEPS model could be used for mapping and updating the spatiotemporal distribution of forest AGB at regional scales driven by multi-source remotely sensed data. The BEPS model is stemmed from the “Farquhar model” [25] which is an instantaneous light response model at leaf level. By simulating the processes of photosynthesis, respiration, and carbon allocation for the forest ecosystem, we could explain the mechanism of every parameter in this model and improve the estimation precision compared with the statistical model used by most of the unisource remotely sensed-based approaches mentioned above. We can also quantitatively evaluate not only the effects of biotic factors such as leaf area index (LAI), but also the effects of abiotic factors such as solar radiation, precipitation, humidity, temperature, and human activities on forest AGB updating by using this physical-based model. The specific goals of this study were to:

(1) Develop an approach to generate the time series of LAI products with fine spatial resolution as important input parameters to drive the BEPS model;
(2) Add a small physical-based module to update the spatiotemporal variations of baseline forest AGB based on the BEPS model, and;
(3) Test and validate the updated forest AGB using the results obtained from field- and ALS-based methods.

## 2. Preparation

### 2.1. Study Area

**Figure 1:**
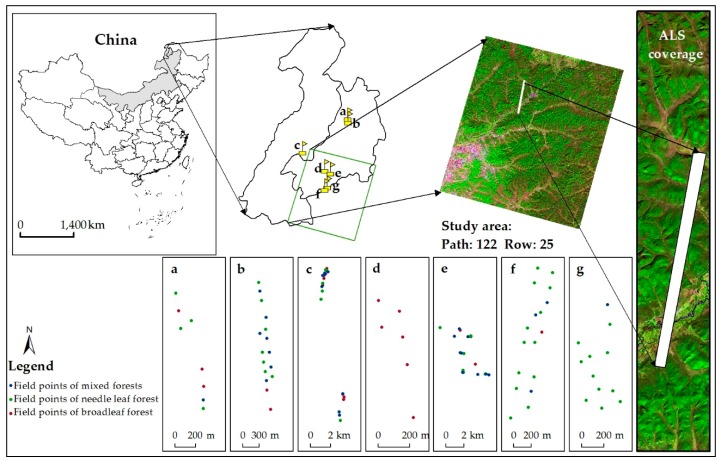
The geolocation of the study area was covered by a pseudo-color composite (R: band 5, G: band 4, B: band 3) Landsat ETM+ image (path: 122, row: 25) for 2012 with overlaid ALS coverage strip; Blue, Green and red points represented field points of mixed forests, needle leaf forest and broadleaf forest, respectively.

The study area was located on the west slope of the Great Khingan Mountains (GKM) extending from northeast to southwest (between 120°23′ E to 122°40′ E and 49°32′ N to 51°15′ N) with elevations ranging from 1100 m to 1400 m (Figure 1). The cold temperate continental monsoon climate led to an annual average temperature of this region as low as −5.0 °C, with maximum and minimum temperatures of 32 °C and −48 °C, respectively. The precipitation of this region was generally unevenly distributed within a year, with about 80–90% of the total annual precipitation (around 400–500 mm) occurring from June to August. The number of days with snow cover was around 200 days, and the growing season lasted 80–100 days. These climate conditions gave this area the typical boreal temperate forest. The forest cover of this region was up to 62% with dominant tree species as Dahurian larch (*Larix gmelinii*), Mongolian Scotch pine (*Pinus sylvestris* L.), white birch (*Betula platyphylla*) and aspen (*Populus davidiana*).

### 2.2. Data

Multi-source data were needed to drive the BEPS model including LAI, baseline AGB, forest types, soil type, carbon dioxide (CO_2_) and meteorological data. The field data and ALS data were used to produce the validation data. We obtained CO_2_ and soil type data from the earth system research laboratory (ERSL) Global Monitoring Division of NOAA (ftp://aftp.cmdl.noaa.gov). Other data were obtained using the methods described in the following subsection.

#### 2.2.1. Field Measurement Data

We conducted field measurements at 56 forest plots (Figure 1a–d,f,g) between 30 August to 15 September in 2012 and at 18 forest plots (Figure 1e) during 10–18 August 2013. These 74 plots consisted of 24 needle leaf forest plots, 21 broadleaf forest plots, and 29 mixed forest plots. The average diameters at breast height (DBH) ranged from 10 cm to 15 cm and the average tree heights ranged from 15 m to 25 m. In each forest plot, we recorded the center location using global positioning system (GPS) [26] working in differential mode, and manually measured the forest structure parameters including LAI, DBH, tree height, and tree crown size in two cross directions for all trees with DBH > 5 cm. The effective LAI (LAIe) of the forest stand was measured using LAI-2200 (Li-Cor, Inc., Lincoln, NE, USA) [27] under diffuse light conditions. We measured forest LAIe at five spatially-balanced locations within a plot and two repeats were made for each measurement, with one above canopy and four below canopy readings using two sets of LAI-2200 with the same type and calibration procedures. For above canopy measurements, measurements were obtained using the LAI-2200 being placed at the open space near forest plots; while for below canopy measurements, the instrument was horizontally held about 1.2 m above the soil background for the sake of reducing the influences of understory vegetation. Then, the LAIe value for each measurement was calculated by using the below and above canopy measurements. At last, the LAIe of a plot was calculated as the average of LAIe measured at five different locations within this plot. The clumping index was obtained using the Tracing Radiation Architecture of Canopy (TRAC, 3rd Wave Engineering, Nepean, ON, Canada) [28] in direct sunlight condition to convert LAIe to true LAI. In addition, we collected data on the environmental conditions of plots including slope, aspect, and elevation.

#### 2.2.2. National Forest Inventory Data

The national forest inventory (NFI) data were compiled by the Chinese Ministry of Forestry in eight periods from 1973 to 2013 with a five-year interval [29]. One inventory was finished in a single year for a specific province and in five years for the whole country, documenting forest areas, types, timber volume and other attributes. The seventh and eighth NFI data in this study area, acquired in 2008 and 2013 respectively, were used to evaluate the forest ABI.

#### 2.2.3. LAI Time Series Data

- MODIS-based LAI time series data

The MODIS reflectance products (i.e., MOD09A1) with 500 m spatial resolution were downloaded from MODIS data center (https://lpdacc.usgs.gov). The MODIS reprojection tool (MRT) was first used to extract red, near infrared, shortwave infrared, solar zenith angle, sensor zenith angle, and the relative azimuthal angle between the sun and sensor information from MOD09A1 data. After converting all maps’ projection into the Universal Transverse Mercator (UTM) projection, we produced the LAI maps with eight-day interval from 2009 to 2012 using the 4-scale geometric optical model by following the method developed by Deng et al. [30]. Then the locally adjusted cubic-spline capping (LACC) algorithm [31] was employed to get the final LAI seasonal curve by removing the outliers.

- Landsat-based LAI time series data

To produce high resolution LAI time series, three Landsat ETM+ images (19 August 2008, 30 August 2012, 2 September 2013) and three Landsat TM images (30 August 2009, 2 September 2010, 5 September 2011) for the Hulun Buir region (path: 122 and row: 25) acquired during the growing season were downloaded from the EROS Data Center (http://earthexplorer.usgs.gov/). Because of the gaps resulting from the scan line corrector breakdown of Landsat ETM+ after May in 2003, we first filled the image gaps using the geo-statistical neighborhood similar pixel interpolator (GNSPI) algorithm based on the spectral information of spatial neighboring pixels [32]. We also conducted geometric and radiative corrections for all Landsat TM/ETM+ images to remove geometric distortions and atmospheric effects [33,34], respectively. In addition, to reduce the atmospheric environment differences among all the Landsat TM/ETM+ images, we conducted the radiative normalization process for these images using the pseudo invariant features (PIFs) (i.e., buildings, roads, or deep water etc.).

After getting all the needed Landsat TM/ETM+images, we first generated the Landsat-based LAI map using the statistical model between the field-based LAI and vegetation index for 2012 and 2013. Based on the MODIS-based LAI seasonal curves and the Landsat-based LAI map for 2012, the eight-day interval time series Landsat-based LAI maps from 2009 to 2012 were produced using the method developed by Wan et al. [35]:

(1) $$T_{\mathrm{LAI},i}=M_{\mathrm{LAI},i}+\frac{46-D_{i}}{46}\times d$$

where *T*_LAI,i_ is the LAI value (m^2^/m^2^) for i^th^ day of a year with 30 m spatial resolution; *M*_LAI,i_ is the LAI value (m^2^/m^2^) for i^th^ day with 500 m spatial resolution for the same forest type corresponding to *T*_LAI,i_; *D_i_* is the date difference (day) between i^th^ day and the nearest day of TM/ETM+-LAI value; *d* is the LAI value (m^2^/m^2^) difference between the predicted date of Landsat TM/ETM+ and the same date of MODIS-LAI. Additionally, the Landsat image of 2008 was also used to produce the baseline AGB to drive the BEPS model.

#### 2.2.4. Forest Types Data

We first produced vegetation-only spatial distribution maps through setting the appropriate Normalized difference vegetation index (NDVI) threshold (i.e., NDVI > 0.3) using Landsat TM/ETM+ images. Then we selected three different categories (i.e., needle leaf forest, broadleaf forest and mixed forests) samples based on the various features such as spectral and texture information of the Landsat TM/ETM+ images using the eCognition (Trimble Navigation Ltd., Westminster, CA, USA) software [36]. The decision tree algorithm was a non-parametric classification technique which outperformed both maximum likelihood estimators and equivalent linear models [37]. Thus, we built the classifier based on the classification rules determined by the decision tree algorithm using See 5.0 software [38]. Finally, we obtained the forest types classification map including broadleaf, needle leaf, and mixed forests by conducting the multi-scale segmentation process in the eCognition environment.

#### 2.2.5. Meteorological Data

We produced the national daily raster images with 500 m spatial resolution by interpolating the meteorological recordings from 753 meteorological observation stations using the Inverse Distance Weighting (IDW) method from 2009 to 2012. The total daily solar radiation was computed by summing up the observed solar duration for every hour during the sun course of a day as Equation (2) [39]:

(2) $$S=1370/10^{6}\cos\theta \times day\;length\times 3600\times (0.1545+0.5489T/day\;length)$$

where *S* is the total daily solar radiation (MJ/m^2^/day); *θ* is the solar zenith angle (degree) for a specific hour during the sun course of a day; *day length* is the daily possible maximum solar duration (hour); *T* is the observed solar duration (hour). In the process, we also quantitatively described the effect of increasing altitude on temperature by assuming that temperature will decrease by 6° for each elevation increase of 1000 m. Then, five meteorological raster layers were generated including daily precipitation (mm), daily maximum temperature (Celsius), daily minimum temperature (Celsius), daily relative humidity (%), and daily solar duration (hour). Finally, corresponding meteorological map layers for our study area were masked based on the boundary vector layer and resampled down to 30 m spatial resolution with the IDW method.

#### 2.2.6. Aerial Laser Scanning Data

ALS data were acquired on 4 and 13 September 2012 at the speed of 180 km/h and height of 1500 m in the clear sky. The ALS system was mounted with laser scanner LMS-Q560 (RIEGL Laser Measurement Systems GmbH, Horn, Australia), GPS, and Inertial Navigation System (INS). The working spectral wavelength was 1550 nm with the beam divergence angle as 0.5 mrad. The recording time interval and the point density were 1 ns and 2 points/m^2^ with the laser pulse frequency as 50 kHz, respectively. The vertical and horizontal accuracy of this ALS system were 0.15 m and 0.50 m, respectively.

## 3. Materials and Methods

**Figure 2:**
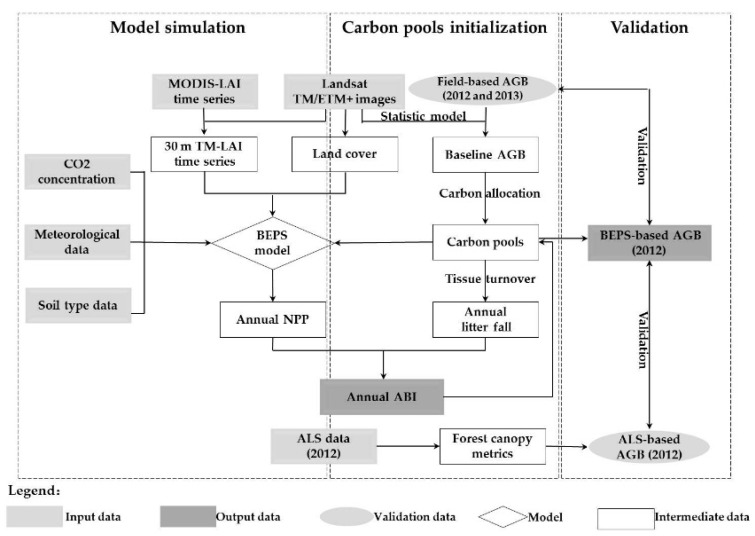
The flowchart of mapping and updating forest AGB by combining multi-source remotely sensed data and ecological process-based model.

This research was carried out in three stages: (1) carbon pools initialization, (2) model simulation and (3) validation stage (Figure 2). In the carbon pools initialization stage, the statistical models were built for different forest types to produce the Landsat-based forest AGB map of 2008 based on the field-based forest LAI and AGB data in 2012 and 2013. Due to the lack of field data of 2008 in the study area, we assumed the empirical relationships between the vegetation index and field-based AGB were universal for the same study area with same forest types, and produced the baseline forest AGB map of 2008 by applying these statistical models. The baseline forest AGB were allocated into different carbon pools of individual trees, including leaf, wood (i.e., stem and branch), and coarse and fine root to parameterize the BEPS model [40]. In the model simulation stage, Landsat-based LAI time series maps with eight-day interval from 2009 to 2012 were generated by combining MODIS-based LAI seasonal variation curves and one available Landsat TM/ETM+ image of each year from 2009 to 2012. The LAI time series images, combined with the forest types, meteorological, soil type, CO_2_ data were all used to drive the ecological process-based BEPS model to conduct the numerical simulation of daily and annual NPP and annual AGB variations. In the validation stage, the forest AGB estimates obtained from ALS and field measurements were used to validate estimates from the BEPS-based method independently.

### 3.1. Forest Aboveground Biomass Mapping

Three kinds of forest AGB results were produced, including field-, Landsat- and ALS-based forest AGBs. Firstly, the field-based forest AGB was produced to build statistical models with the field based-LAI for different forest types. Based on these models and the Landsat-based LAI map, the Landsat-based AGB map of 2008 was created and served as the baseline AGB in the BEPS model to initialize the carbon pools. Finally, both the field- and ALS-based AGB worked as validation data to evaluate the BEPS result.

- Field-based forest aboveground biomass

At the individual tree level, we computed the biomass of leaf (*W*_L_), stem (*W*_S_), and branch (*W*_B_) components of four different tree species (i.e., larch, pine, birch and aspen) based on the one or two factor allometric equations as Equations (3) and (4):

(3) $$W_{i}=a\times {\left(D^{2}H\right)}^{b}$$

(4) $$W_{i}=a\times D^{b}$$

where *W*_i_ is the biomass (kg/m^2^) for leaf (i = L), stem (i = S), and branch (i = B), respectively; a and b are the coefficients; *D* and *H* are the DBH (cm) and tree height (m) of the individual tree. We then obtained total aboveground biomass of an individual tree by summing up the biomass of different aboveground components of an individual tree which calculated by 12 different allometric equations collected from existing published literatures [41,42] (Table 1). At the forest plot level, the total forest AGB was achieved by summing up the AGBs of individual tree within the forest plot.

- Landsat-based forest aboveground biomass

Soil adjusted vegetation index (SAVI), which was proposed by Huete [43] in 1988, was less sensitive to soil influence compared to other vegetation indexes. So, we produced the LAI map of 2012 based on the statistical model between field-based LAI and SAVI [43,44]. Accordingly, the forest AGB map of 2012 was generated using the statistical relationship between forest field-based LAI and AGB at the plot level [45,46]. Assuming that the statistical model between field-based LAI and AGB for the same study area with same forest types was identical in different years, the Landsat ETM+ image-based AGB map of 2008 was generated, and further served as the base map to update forest AGBs for each year from 2009 to 2012.

- ALS-based forest aboveground biomass

Based on the ALS data which explicitly contained the 3-D forest canopy structural information, we first filtered non-ground points and generated the normalized canopy surface model (NCSM) with the grid size as 1 m × 1 m after removing topographic effect using the digital elevation model (DEM) [47,48]. Then, the first return point cloud data, which were considered as the appropriate proxy to estimate forest AGB [49], were used to build the statistical model with forest plot AGB. Among each forest plot, only the canopy points (i.e., height > 2 m) were used to compute lidar metrics, which would be correlated with field-based forest AGB using 9 different percentile heights, including 10% (h_10_), 20% (h_20_), 30% (h_30_), …, 90% (h_90_), canopy mean height (h_m_) and density (d). Finally, all lidar metrics served as independent variables and were inputted into the stepwise regression analysis to build the best statistical model and predict the forest AGB at the landscape level.

### 3.2. Forest Aboveground Biomass Updating

#### 3.2.1. Net Primary Productivity Simulation

To drive the BEPS model, different layers of the raster images including LAI, baseline forest AGB, forest cover types, relative humidity, precipitation, temperature, daily solar radiation and soil data were resampled to the same spatial resolution (30 m) using the IDW method and converted to the same projection (i.e., UTM). With these spatially explicit input data on vegetation, soil, and meteorology, BEPS can be run pixel by pixel over a defined domain. By simulating the processes of photosynthesis, respiration, and carbon allocation for the forest ecosystem, which were determined by the input parameters, the BEPS model can be used for updating forest AGB [24,50]. Prior to the actual simulation, the proper initialization of various carbon pools was required. It was then possible to alter the size of a given carbon pools based on the lost (decomposed) or added (from other carbon pools) carbon [40]. For the first day, carbon value for a given carbon pool was the value obtained from the baseline forest AGB. Then the numerical simulation was conducted at daily time step for various ecological processes, such as photosynthesis, respiration, carbon allocation, water cycle and energy balance [24]. In addition, sunlit and shaded leaves were separated in BEPS to better approximate real photosynthesis process. Therefore, the daily net assimilated carbon by plants (i.e., NPP) (gC/m^2^) can be obtained by subtracting energy consumed by the respiration process from gross primary productivity (GPP) (gC/m^2^) as Equation (5):

(5) $$NPP=GPP-R_{g}-R_{m}$$

where *R*_g_ is the respiration consumed by plant growth process (gC/m^2^); *R*_m_ is the plant maintaining respiration (gC/m^2^). *R*_g_ is assumed as 25% of GPP while *R*_m_ is calculated as the summation of maintenance respiration of leaf, wood, and fine and coarse roots [51]. The maintenance respiration of individual vegetation pools is estimated according to pool sizes, maintenance respiration rates at the base temperature, and air temperature.

#### 3.2.2. Updating Forest Aboveground Biomass

The ABI over a certain time can be obtained by subtracting the amount of litter fall and dead matter from the total NPP over the time period. Based on this definition of the forest ABI, the *ABI*_j_ can be computed as:

(6) $$ABI_{j}=2\times \sum_{i}\left[NPP_{j}\times A_{i}\times (1-T_{i})-B_{i,j-1}\times T_{i}\right]$$

where i denotes the different plant components such as stem, leaf and branch; A_i_ is the carbon allocation coefficient (%) for different carbon pools; *T* is the carbon turnover rate (%) between different carbon pools; *B*_(i,j−1)_ is the AGB values (kg/m^2^) of different plant components in the (j − 1)^th^ year; the multiplication factor of 2 in the right side of Equation (6) means that the carbon content factor is 0.5 [52]. Then, the updated forest AGB may be computed based on the baseline forest AGB and the ABI over a certain time as Equation (7):

(7) $$AGB_{j}=AGB_{j-1}+ABI_{j}$$

where *AGB*_j_ is the updated forest AGB (kg/m^2^) in the j^th^ year; *AGB*_j−1_ is the baseline forest AGB (kg/m^2^) for the (j − 1)^th^ year; and *ABI*_j_ is the forest AGB increment (kg/m^2^) during the j^th^ year. The Landsat-based forest AGB of 2008 was used as the baseline forest AGB and being allocated into different carbon pools of an individual live standing tree according to the allocation coefficients obtained from our previous work and published literature [24,53] (Table 2).

### 3.3. Accuracy Assessment

The Landsat TM/ETM+ image-based forest types classification map was assessed with 256 sample points selected based on both site observation and aerial photo visual interpretation. The kappa coefficient (K) was provided as an indicator to assess the classification accuracy and computed as:

(8) $$K=\frac{N\sum_{k}x_{\mathrm{kk}}-\sum_{k}x_{k\sum x\sum k}}{N^{2}-\sum_{k}x_{k\sum x\sum k}}$$

where N is the total number of pixels in all the ground truth classes; k is the dimension of confusion matrix which used to show the accuracy of a classification result; *X* represents a pixel value.

In addition, the BEPS-based ABI result was compared with one computed from the NFI-based method. Due to the fact that NFI data were collected every five years [54], these data cannot directly predict the annual forest ABI nor validate the BEPS-based annual forest ABI result. Moreover, there was no other suitable data which could verify the annual ABI. Therefore, we validated the five years’ ABI by computing the NFI-based AGB difference based on the seventh and eighth NFI data with a five-year time gap. Finally, both field- and ALS-based forest AGB estimates were used as independent data to assess the BEPS-based updated forest AGB result for 2012.

## 4. Results

### 4.1. Forest Types Map

**Figure 3:**
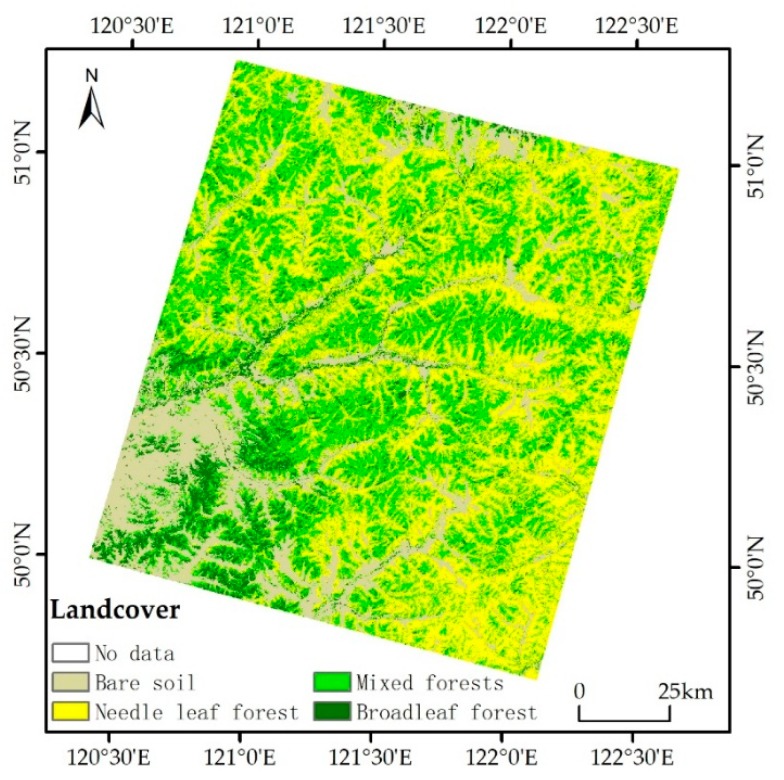
Landcover map for 2012.

We classified the Landsat ETM+ images of 2008 and 2012 into three different forest types including broadleaf forest, needle leaf forest, and mixed forest types (Figure 3). Using the landcover map for 2012 as an example, by building the confusion matrix to compare the computer-based classification with manually selected 256 validation samples of different topography and forest types, we found that the producer’s accuracy for three forest categories were better than 80% while a little lower for the non-forest category (77.8%). The overall accuracy for the whole study area was 84.0% with kappa coefficient of 0.76.

### 4.2. Forest Aboveground Biomass Map

#### 4.2.1. Landsat-Based AGB of 2008

The eight-day interval LAI maps with 30 m spatial resolution for years from 2009 to 2012 were generated as important input parameters to drive BEPS model. The statistical relationships between the forest field-based LAI and SAVI from Landsat ETM+ images of 2012 and 2013 were strong for all forest types including needle leaf forest (R^2^ = 0.72, *n* = 24, *p* < 0.01), broadleaf forest (R^2^ = 0.57, *n* = 21, *p* < 0.01), and mixed forests (R^2^ = 0.58, *n* = 29, *p* < 0.01). The LAI for 2012 in the study area varied from 0.1 to 4.80 with average value and standard deviation of 1.60 (m^2^/m^2^) and 0.62 (m^2^/m^2^), respectively.

- After assuming that the empirical relationship between the field-based LAI and AGB was universal with the same remote sensor, sampling season and study area in different years, we generated the forest AGB map of 2008 based on the statistical model built using the forest field data collected in the summer of 2012 and 2013.
- As shown in Table 3, the field-based LAI was used as independent variable to build the statistical model with field-based forest AGB at forest plot level to produce Landsat-based forest AGB map. The needle leaf forest had the best correlation coefficient (R^2^ = 0.72, *n* = 24, *p* < 0.01), and the Landsat TM/ETM+ image-based LAI of broadleaf forest and mixed forests explained 42% (*n* = 21, *p* < 0.05) and 57 % (*n* = 29, *p* < 0.01) of variations in field-based forest AGB, respectively. Finally, the forest AGB map of 2008 based on the developed model was generated and served as the baseline data to parameterize the ecological process-based model to simulate the annual AGB variations.

#### 4.2.2. ALS-Based AGB of 2012

**Figure 4:**
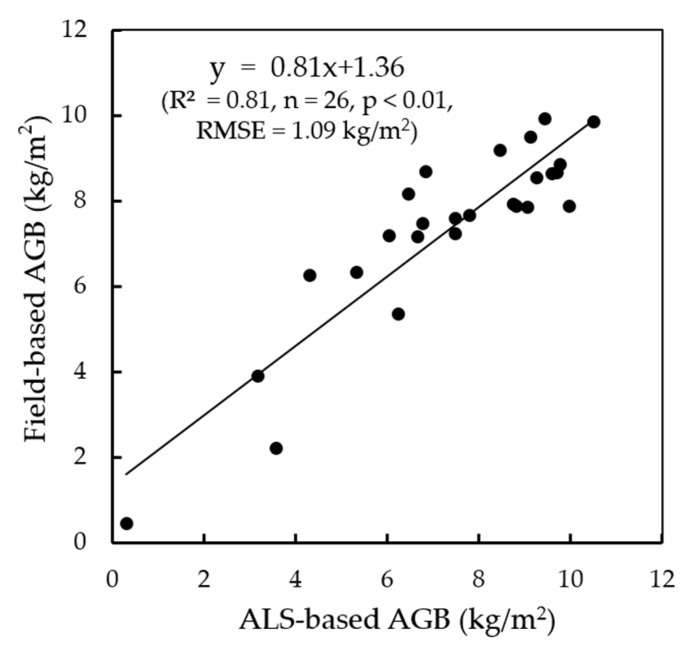
The validation of ALS-based forest AGB in 2012 using the results obtained from forest field-based AGB at the plot level.

- We built the stepwise regression model with the field-based forest AGB as a dependent variable by inputting various ALS-based canopy metrics including nine canopy percentile heights, mean height and plot density (Table 4) as independent variables. It was found that the canopy mean height did a good job in predicting the variations in field-based forest AGB with the linear regression model as *AGB* = 0.826 × *h*_m_ − 3.208 (R^2^ = 0.83, *n* = 26, *p* < 0.01, RMSE = 1.09 kg/m^2^).
- After getting the ALS-based forest AGB map, we found that it had a heterogeneous spatial distribution pattern with maximum, minimum, and average values of 16.27 kg/m^2^, 0.06 kg/m^2^, and 6.07 kg/m^2^, respectively. By comparing the forest AGB of 2012 between the ALS- and field-based methods, high correlation (R^2^ = 0.81, *n* = 26, *p* < 0.01) was observed (Figure 4), which showed that the ALS-based forest AGB could serve as a validation data for the BEPS results.

### 4.3. Forest Aboveground Biomass Update

#### 4.3.1. Carbon Pools Initialization

The Landsat-based AGB (*x*) (kg/m^2^) values effectively captured the variation of wood biomass (*y*_s_) (kg/m^2^) with the linear regression statistical model as *y*_s_ = 0.985*x* − 0.049 (R^2^ = 0.99, *n* = 35, *p* < 0.001). Furthermore, it predicted 93.8% of variation in the coarse root biomass (*y*_r_) with the exponent regression statistical model as *y*_r_ = 0.293*x*^1.051^ (*n* = 35, *p* < 0.001) and explained 87.3% of variation in the leaf biomass (*y*_l_) with the exponent model as *y*_1_ = 0.032*x*^0.810^ (*n* = 35, *p* < 0.001). Based on these three statistical models, the wood, leaf, and root carbon pools were initialized by determining the coefficients of wood, leaf, and root biomass to AGB from the above models. Assuming the fine root carbon pool and the leaf carbon pool were similar [55,56,57,58], the initial fine root carbon pool can also be determined.

#### 4.3.2. GPP, NPP, and AGB Variations

We obtained the mean GPP, NPP, and AGB of different forest types from 2009 to 2012 based on the daily and annual simulated outputs from the BEPS model. It was found that the total amount of GPP, NPP and AGB showed a general annual increasing pattern for all forest types from 2009 to 2012. The GPP, NPP and AGB between 2010 and 2011 had the maximum annual increases for all three different forest types. The annual mean GPPs were computed for needle leaf forest (i.e., 1322 gC/m^2^), broadleaf forest (i.e., 1319 gC/m^2^), and mixed forests (i.e., 1613 gC/m^2^), respectively (Table 5). The annual mean NPP and ABI values were also shown in Table 5. In terms of GPP of the needle leaf forest, it continued to increase with the growth rate as 42.39 gC/m^2^/yr (year), while rate of tree growth for the broadleaf forest and mixed forests were 37.87 gC/m^2^/yr and 40.47 gC/m^2^/yr, respectively. The NPP of mixed forests had the maximum tree growth rate of 14.97 gC/m^2^/yr and the broadleaf forest had the minimum rate of growth of 13.82 gC/m^2^/yr. The average annual ABI of needle leaf and mixed forests were 0.16 kg/m^2^/yr and 0.20 kg/m^2^/yr, respectively, while the broadleaf forest AGB only increased 0.14 kg/m^2^/yr.

We obtained the NFI-based accumulated ABI value between the seventh and eighth NFI data as 0.12 kg/m^2^, 0.47 kg/m^2^, 0.63 kg/m^2^, and 1.16 kg/m^2^ for the Eergu, Genhe, Yake, and Elunchn counties, respectively. Then, the mean ABI for our study area was 0.61 kg/m^2^ after using the ratio of area of forested pixels in each county over the whole Landsat images’ forested area during five years. The BEPS-based mean annual ABI over our study area during years from 2008 to 2012 was 0.16 kg/m^2^ which was similar but slightly higher than NFI-based annual mean ABI (0.12 kg/m^2^) between the seventh and eighth NFI data.

#### 4.3.3. Updated Forest Aboveground Biomass

**Figure 5:**
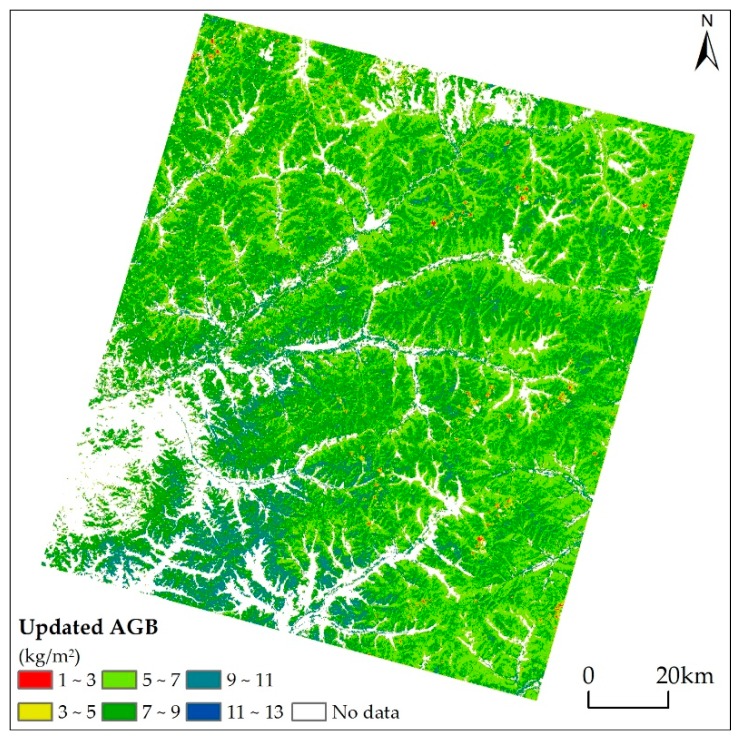
The BEPS-based forest AGB for 2012.

The predicted forest AGB of 2012 (Figure 5) was obtained based on the annual ABIs ranging from 2009 to 2012 and the baseline forest AGB of 2008. The forest AGB values ranging from 5 to 9 kg/m^2^ accounted for 81.89% of this study area. 

**Figure 6:**
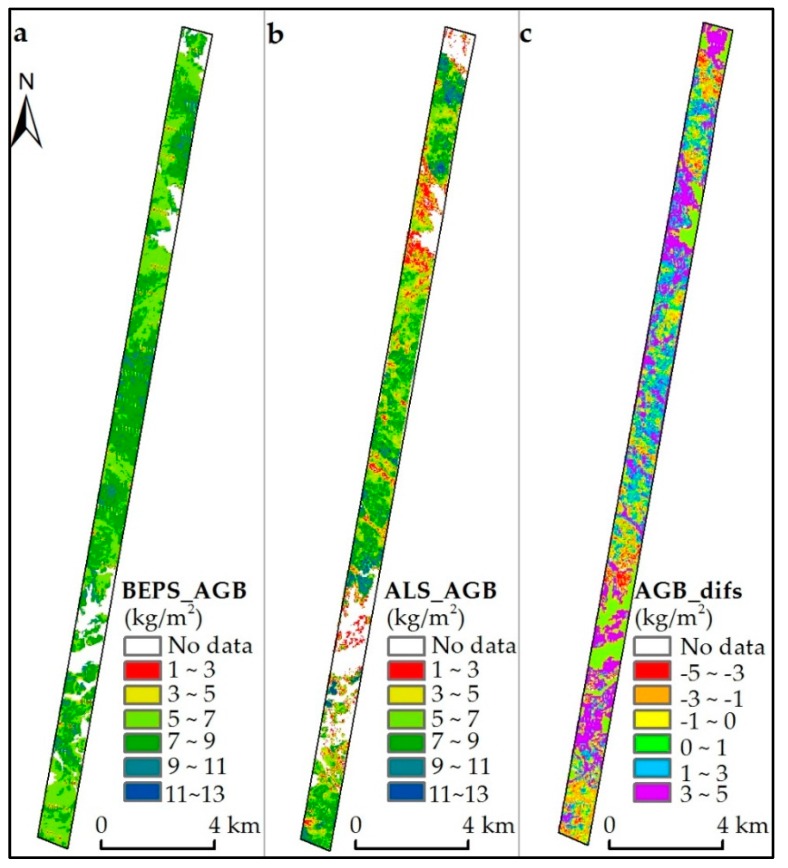
The spatial distribution maps of BEPS-based (**a**), ALS-based (**b**) forest AGB for 2012 and the differences map between BEPS- and ALS-based maps (**c**).

The cross-comparison between the predicted forest AGB and the ALS-based forest AGB of 2012 (Figure 6a,b) found that the consistent spatial distribution pattern could be observed in both forest AGB maps. The areas with high or low values of forest AGB from the BEPS-based AGB map could also be observed in the ALS-based estimates. However, the ALS-based AGB tended to over- and under-estimate the results obtained using the BEPS-based method in the high- and low-AGB areas. From the AGB differences map (Figure 6c), we found that most of the differences were between −3 kg/m^2^ and 3 kg/m^2^ except in areas where tree height was taller than 2 m. 

**Figure 7:**
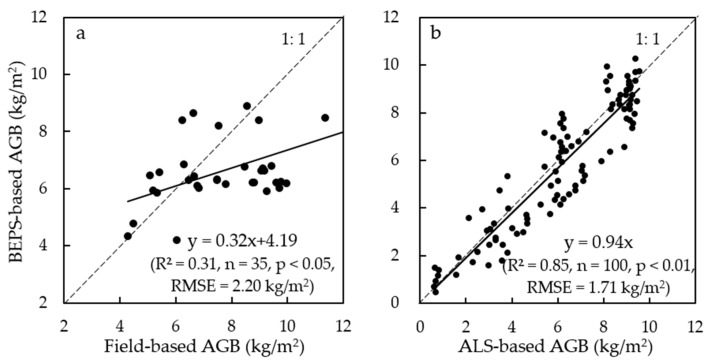
The validation of BEPS-based forest AGB in 2012 using the results obtained from forest field-based AGB (**a**), and ALS-based AGB (**b**) at the plot level.

Although the BEPS model tended to overestimate forest AGB which could have resulted from the overestimated NPP simulated by BEPS model, this BEPS model-based AGB map accounted for 31% of variation in the field-based forest AGB (R^2^ = 0.31, *n* = 35, *p* < 0.05) (Figure 7a). Also, it could effectively capture the overall variation in the ALS-based forest AGB (R^2^ = 0.85, *n* = 100, *p* < 0.01) (Figure 7b).

## 5. Discussion

In this work, we have three major discoveries: (1) It is possible to generate fine spatial (i.e., 30 m) LAI products with eight-day intervals by combining MODIS and TM data. (2) The BEPS model could be used to update the spatiotemporal distributions of forest AGB driven by data on vegetation, meteorology, and soil. (3) When compared with field- and ALS-based forest AGB, we found that the BEPS-based AGB could capture variations of validation data well.

### 5.1. Landsat Radiative Normalization

**Figure 8:**
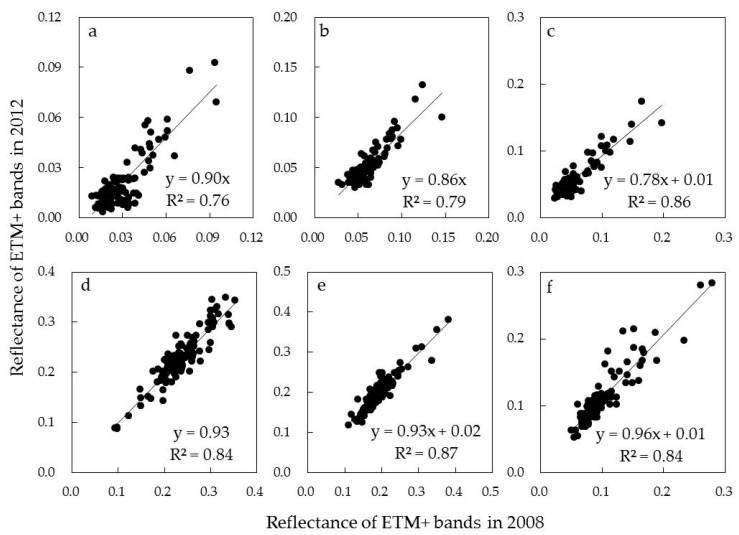
The comparisons between the reflectance values of the PIFs objects for band 1 (**a**), band 2 (**b**), band 3 (**c**), band 4 (**d**), band 5 (**e**) and band 6 (**f**) of Landsat ETM+ images in 2008 and 2012.

As shown in Figure 8, the reflectance values of PIFs from the Landsat images of 2008 and 2012 were compared from band 1 to band 6. The linear regression models were built for each band, and the correlation coefficients were 76%, 79%, 86%, 84%, 87% and 84%, respectively. The significant correlation between the reflectance values of the same band in different time confirmed that the radiative correction and radiation normalization for Landsat images were done correctly and successfully. It provided a solid basis on which to apply the empirical model developed with the forest field AGB and the spectral information of the ETM+ image in 2012 to the same area with same forest types in 2008. However, because the spectral response of TM/ETM+ images were not linear with the increasing of forest AGB, we recommend incorporating other information, such as forest age, to further improve the accuracy of forest AGB estimation.

### 5.2. Uncertainties in LAI Time Series Images

We produced the MODIS-based LAI seasonal variation curves for different forest types with spatial resolution of 500 m. The different spatial resolutions of MODIS (500 m) and Landsat TM/ETM+ (30 m) images might result in different LAI values in the same location due to the mixed pixels in MODIS images, even after applying the smooth interpolation procedures. Moreover, clumping index is a key parameter to retrieve LAI based on the MODIS data, which is closely related with forest types. The different forest types might result in different MODIS-based LAI estimates. Finally, the noisy signals of MODIS time series images, such as cloud, would affect the LAI seasonal variation curves for a specific forest type. In addition, gaps on raw Landsat images were filled using the GNSPI algorithm [32]. The uncertainties in the filled spectral values would be definitely propagated into the retrieved LAI.

### 5.3. Updated Forest Aboveground Biomass

It was shown in Figure 5 that the forest AGB was mainly distributed in the southern part of Genhe County and northern part of Yake County. A more detailed spatial distribution pattern can be found in the ALS coverage area due to its high spatial resolution. When comparing the BEPS- with ALS-based AGB in 2012 (Figure 6), although they showed a similar distribution pattern, ALS-based estimates were much lower compared with the corresponding low value regions in the BEPS-based results. This was because only the trees whose heights were larger than 2 m were used from the ALS data to compute forest AGB and led to ALS data being unable to capture low AGB value regions. For the high value regions, the ALS-based AGB were slightly higher than BEPS-based results. This might be attributed to the saturation problem for the optical remotely sensed images when used for LAI inversion. Another reason may result from the different accuracy and spatial resolution of land cover maps obtained from Landsat data and ALS data. The Landsat-based land cover was produced using the supervised classification by manually selected training samples, while the ALS-based land cover was produced using the category information delivered by the vendor. In fact, the validated spatially continuous ALS-based AGB map can serve as a “bridge” for validation and comparison between the AGB results obtained from coarse remotely sensed data and field-based results at the forest plot level.

The BEPS-based annual mean forest AGB was 7.13 kg/m^2^ for the period from 2008 to 2012, which was similar to but slightly larger than the result (i.e., 6.25 kg/m^2^) obtained in the same forest region by another research [59]. This might be due to the older measurements used in their work (year 2003). During the period between two field-based data, forest AGB would increase due to the strict forest protection policy in our study area. In addition, Mao et al. [60] found that most Landsat TM-based forest AGB values were close to level of 6–10 kg/m^2^ in the northeastern part of China after 2000. Meantime, by combing optical and SAR data, Shao et al. [61] found that the biomass values were mainly distributed over the range from 7 to 12 kg/m^2^ in Genhe, China for 2013. Their results were similar to and further testified the results in this research.

### 5.4. Uncertainties in Updated Forest Aboveground Biomass

When comparing forest AGBs obtained using the BEPS-based method with the one produced based on with field-based measurements, the discrepancy between them was distinguishable even if the determination coefficient between them was statistically at a significance level of 0.05. The BEPS-based AGB was mostly higher than field-based AGB for plots with low field-based AGB. In contrast, the BEPS-based AGB was mostly lower than field-based AGB for plots with high field-based AGB (Figure 7).

**Figure 9:**
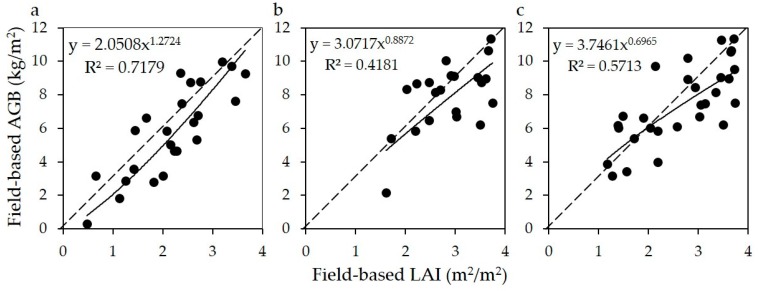
The relationship between field-based LAI and AGB. Inset (**a**–**c**) represented needle leaf, broadleaf and mixed leaf forests, respectively.

Uncertainties in BEPS-based AGB results were mainly from two factors. The first factor was the initialization uncertainties of AGB in 2008, which was estimated using the AGB-LAI empirical model set up with field measurements recorded in 2012. As shown in Figure 9, AGB-LAI empirical model tended to underestimate or overestimate high or low AGB, respectively. The second one was the uncertainties in annual NPP simulated by the BEPS model, which was mainly driven by the LAI series generated by the MODIS and TM data. When estimating the remotely sensed-based LAI with the empirical relationship between field-based LAI and vegetation index calculating from remote sensing images, it would cause underestimation or overestimation of overstory LAI for dense or sparse forests [62], respectively. The underestimation of high LAI was mainly caused by the spectral saturation of optical remote sensing [62] while the overestimation of low AGB mainly resulted from the large contribution of understory vegetation to the reflected signal detected by the sensor [63]. The saturation of optical remote sensing signals and contribution of understory vegetation caused underestimation or overestimation of overstory LAI for dense or sparse forests, respectively, which induced annual NPP and ABI of overstory to be underestimated or overestimated for dense or sparse forests, respectively. Of course, uncertainties in field-based AGB also have influences on the agreement between BEPS-based and field-based AGB. Field-based dataset from 74 plots is not large enough, although we set up these plots with different density categories of forest canopy and different topography variations under the guidance of local forest explorers. These plots were distinguished by three classes (i.e., needle leaf forest, broadleaf forest, and mixed forests) when used for building models for LAI and AGB estimations. The limitation in the number of sampling plots would potentially induce some uncertainties in the trained statistical model. Undoubtedly, the accuracy of the field-based LAI and AGB results calculated from these models would be affected.

Differences between the BEPS- and ALS/field-based AGBs could also be introduced from other three aspects, including statistical model, landcover maps and forest ages. Due to the lack of field measurements in 2008, AGB in 2008 was estimated from remotely sensed LAI with the statistical model trained using field measurements of AGB and LAI taken in 2012 and 2013. This simple temporal extrapolation of the statistical model might induce uncertainness in the baseline forest AGB of 2008, which will be propagated into updated BEPS-based AGB.

Additionally, errors were also brought from landcover maps. (1)The Landsat ETM+ image-based forest cover maps only for 2008 and 2012 were produced to characterize the forest dynamic changes, which might not adequately capture the annual variations of forest types between neighboring years. (2) The different land surface objects of a forest stand measured by remote sensors and field measurements work may introduce errors. The information collected by MODIS or Landsat TM/ETM+ contained the spectral responses of vertical structure of a forest stand including overstory (i.e., live standing trees), understory (i.e., shrubs, grass) [64], bare soil ground, and the shadows between the forest canopies. However, the forest AGB measured by field work only concerned the live standing trees with DBH >5 cm. Thus, the discrepancy between the field- and passive remotely sensed data-based forest AGBs might be improved through removing the forest background reflectance influence. The contribution of forest background (including shrubs and grass and soil etc.) to forest canopy reflectance has been successfully and quantitatively characterized by combining the multi-angle reflectance observation and geometric optical model [63,65]. However, the effects of different background (i.e., bare soil only, shrubs and grass, or soil + shrubs) on forest AGB estimation is still under discussion which is a direction deserved for further research. (3) The errors introduced by broad forest cover type maps could also have an impact on the forest updated AGB result. We should have used the tree species dependent carbon allocations and turnover coefficients of carbon pools initialization, but forest cover type had only been classified as broadleaf, needle leaf, and mixed forests types in this study. The lower levels of detail in broad forest types might diminish the heterogeneous of the forest AGB variations and spatial distribution patterns.

Finally, the trees with different ages will show different photosynthesis rates, even at the same environment conditions. Thus, the forest age should be considered to differentiate the simulated NPP of forest trees. However, forest age mapping from the passive remotely sensed data is a challenging question, and this deserves further research.

### 5.5. Management Implications and Future Researches

Forest managers are often concerned about the ability of ecosystems to sequester and store carbon. Long-term net flux of carbon between terrestrial ecosystems and the atmosphere have been dominated by changes in both forest area and biomass that result from management and regrowth [52]. The process presented in this study which provides a tool for understanding rates of forest AGB accumulation at small temporal scales can provide managers with valuable information about the carbon dynamics of an ecosystem.

In addition, this model may provide a basis to understand how ecosystems respond to disturbance, such as wildfire, as well as estimate their potential to emit carbon to the atmosphere. Wildfires can consume substantial proportions of AGB in some ecosystems [66,67], and estimates of AGB may offer a basis on which to approximate potential emissions from fire. Moreover, quantifying the accumulation of AGB due to regrowth after wildfire can provide insights into the recovery of forest ecosystems and the key factors regulating biomass accumulation [68].

This research can help identify factors that drive accumulation of AGB in a forest ecosystem, which will improve our understanding of how changes in climate and fire regimes affect the carbon balance [68]. Future research is needed to address where AGB accumulation is occurring in forest ecosystems, as there are considerations for forest management such as managing wildfire hazards, depending on the strata (for example: ladder fuels or upper canopy) where AGB is amassing.

## 6. Conclusions

In this work, we developed an approach to map and update forest AGB by combining the multi-source remotely sensed data and a process-based ecological model, and concluded that:

1. The process-based model driven by multi-source remotely sensed data could be used to dynamically forecast and update the forest AGB at the landscape level. The BEPS-based results explained 31% of variation in the field-based AGB and 85% of variation in the ALS-based AGB with the confidence level higher than 95%.
2. Both forest biotic (i.e., LAI, forest type etc.) and abiotic factors, such as soil type data and meteorological data, were considered in predicting the spatiotemporal distribution of forest AGB through the process-based ecological model.
3. Since the Landsat-based AGB predicted 99.0%, 93.8%, and 87.3% of variation in the wood biomass, coarse root biomass and leaf biomass, respectively, the initial forest AGB spatial distribution map can be used to parameterize the forest carbon pools quantitatively characterized in the process-based model.

The dynamic and timely spatiotemporal distribution maps of updated forest AGB will be beneficial to the long-term monitoring for ecological observational studies and quantitatively assessing the carbon sequestration of forest ecosystem.

## Figures and Tables

**Table 1:** The allometric equations for biomass estimation of an individual tree’s different components.

Species	Stem Biomass	Branch Biomass	Leaf Biomass	Source
**Larch**	*W*_s_ = 0.0461 × (*D*^2^*H*)^0.8722^	*W*_B_ = 0.0356 × (*D*^2^*H*)^0.5624^	*W*_L_ = 0.0140 × (*D*^2^*H*)^0.5628^	[41]
**Pine**	*W*_s_ = 0.3364 × *D*^2.0067^	*W*_B_ = 0.2983 × *D*^1.144^	*W*_L_ = 0.2931 × *D*^0.8486^	[42]
**Birch**	*W*_s_ = 0.0494 × (*D*^2^*H*)^0.9011^	*W*_B_ = 0.0142 × (*D*^2^*H*)^0.7686^	*W*_L_ = 0.0110 × (*D*^2^*H*)^0.6472^	[41]
**Aspen**	*W*_s_ = 0.2286 × (*D*^2^*H*)^0.6933^	*W*_B_ = 0.0247 × (*D*^2^*H*)^0.7378^	*W*_L_ = 0.0108 × (*D*^2^*H*)^0.8181^	[41]

Note: Here *D* is the DBH (cm); H is the tree height (m); *W*_s_ is the stem biomass (kg/m^2^); *W*_b_ is the branch biomass (kg/m^2^); *W*_L_ is the leaf biomass (kg/m^2^).

**Table 2:** Carbon allocation coefficients (CAC) and turnover rates (TR) used in the process-based BEPS model.

Parameters	Needle Leaf Forest	Broadleaf Forest	Mixed Forests
**Wood CAC**	0.301	0.462	0.382
**Leaf CAC**	0.213	0.223	0.208
**Coarse root CAC**	0.148	0.119	0.154
**Fine root CAC**	0.348	0.196	0.257
**Wood TR**	0.028	0.029	0.028
**Leaf TR**	1.000	1.000	1.000
**Coarse root TR**	0.027	0.045	0.027
**Fine root TR**	0.595	0.595	0.595

**Table 3:** The statistical models for forest field-based forest AGB and LAI for different forest types.

Forest Type	Sample No.	Statistical Models	R^2^	*p* <
**Needle leaf**	24	$AGB=2.0508\times LAI^{1.2724}$	0.72	0.01
**Broadleaf**	21	$AGB=3.0717\times LAI^{0.8872}$	0.42	0.05
**Mixed**	29	$AGB=3.7461\times LAI^{0.6965}$	0.57	0.01

**Table 4:** The correlation analysis between field-based AGB and ALS-based canopy metrics at forest plot level.

ALS Metrics	h_m_	d	h_10_	h_20_	h_30_	h_40_	h_50_	h_60_	h_70_	h_80_	h_90_
**AGB**	0.91	0.56	0.78	0.85	0.87	0.88	0.89	0.89	0.88	0.88	0.87

Note: Here AGB is the forest aboveground biomass (kg/m^2^); *h*_m_ is the mean canopy height (m); d is forest plot density (trees/hm^2^); h_10_, h_20_, …, h_90_ are the 9 different percentile heights computed based on the ALS-based point cloud data.

**Table 5:** Annual mean GPP, NPP and AGB values and their annual mean increment of different forest types from 2008 to 2012.

Forest Type	GPP (gC/m^2^)	NPP (gC/m^2^)	AGB (kg/m^2^)	Increment of GPP (gC/m^2^/yr)	Increment of NPP (gC/m^2^/yr)	Increment of AGB (ABI) (kg/m^2^/yr)
Needle leaf	1322	672	6.29	42.39	13.82	0.16
Broadleaf	1319	863	8.01	37.87	14.81	0.14
Mixed	1613	679	7.88	40.47	14.97	0.20
